# Stereotactic Vacuum-Assisted Minimally Invasive Aspiration of Hemorrhagic Stroke

**DOI:** 10.7759/cureus.23706

**Published:** 2022-03-31

**Authors:** Gyusik Park, Tijil Agarwal, Arthur Wang, Ninh Doan

**Affiliations:** 1 Neurology, University of Alabama at Birmingham School of Medicine, Birmingham, USA; 2 Neurology, Baptist Medical Center South, Montgomery, USA; 3 Neurosurgery, Tulane University School of Medicine, New Orleans, USA; 4 Neurosurgery, Baptist Medical Center South, Montgomery, USA

**Keywords:** brain injury, stroke, minimally invasive surgery, hematoma evacuation, intraparenchymal hemorrhage, hemorrhagic stroke, intracerebral hemorrhage

## Abstract

Intracerebral hemorrhage (ICH), accounting for 9-27% of all strokes, carries substantial rates of morbidity and mortality that have not shown much improvement in the past decades. The poor outcomes of ICH can be attributed to the primary and secondary brain injuries caused by mass effects and inflammatory mechanisms, respectively. Early ICH evacuation is a critical component of treatment, as it mitigates the effect of both the primary and secondary mechanisms of brain injury and is associated with significant improvement in patient outcomes. However, no standardized evacuation technique exists. This technical report introduces a novel stereotactic vacuum-assisted minimally invasive (MIS) aspiration of a hemorrhagic stroke with its effectiveness evidenced by excellent patient recovery.

## Introduction

Intracerebral hemorrhage (ICH) accounts for 9-27% of all strokes, is the second most common after ischemic stroke, and carries substantial morbidity and mortality rates [1]. Despite major advances in clinical neurology and the significant decline in mortality rates following ischemic stroke, survival following hemorrhagic stroke showed minimal improvement in the past decades [2]. Hemorrhagic stroke continues to be one of the leading causes of death with a one-year survival rate of around 35-49%, with half of the deaths occurring within the first two days after onset [3]. Even when patients survive, only 20% of them are expected to be independent at six months due to permanent disability from neurological and cognitive deficits [4].

Due to the complex and unclear pathophysiological process of neuronal death in ICH, treatment and management are very limited. Surgical interventions include craniotomy and open surgery to remove hematomas, decompressive craniectomy, minimally invasive aspiration using a catheter, external ventricular drain (EVD) for intraventricular hemorrhage (IVH), and more [5]. These approaches have limited success. We describe here a novel stereotactic vacuum-assisted (MIS) aspiration of a hemorrhagic stroke in a patient who regained most of his motor and speech functions after suffering from a large, left-sided basal ganglia hemorrhagic stroke.

## Technical report

**Figure 1:**
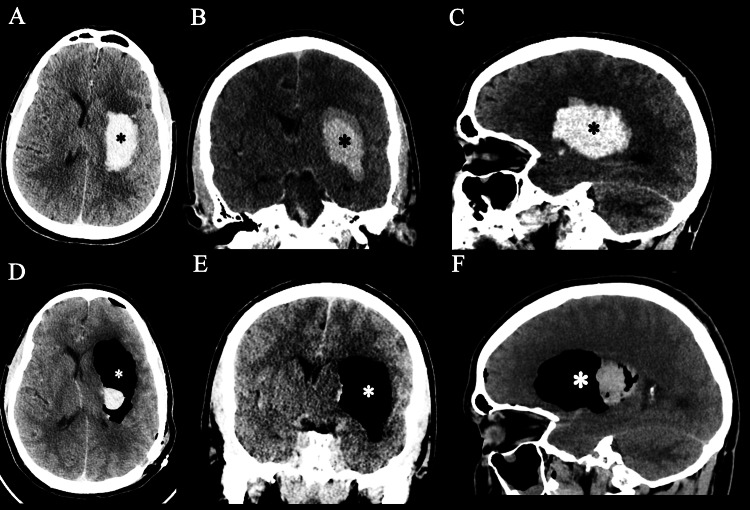
Preoperative CT brain images (A, B, C) showing a large basal ganglia hemorrhage (*) involving the globus pallidus and the internal capsule, and postoperative CT brain images (D, E, F) showing the nearly complete evacuation of the hematoma with the space being replaced with pneumocephalus (*) and a small residual hematoma

A 37-year-old man with a past medical history of hypertension presented to the emergency department with right-sided weakness, profuse vomiting, and minimal responsiveness. On arrival, the patient was intubated for airway protection. Vital signs on admission were significant for blood pressure of 233 mmHg systolic and 172 mmHg diastolic. The patient had dense right-sided hemiplegia and did not follow any commands. Glasgow Coma Scale (GCS) on admission was 3T. CT brain on admission showed an acute left basal ganglia intraparenchymal hematoma (6 x 5 x 3 cm) with a small mass effect on the left lateral ventricle and a 5 mm left-to-right subfalcine midline shift (Figures 1A-1C). CT angiography of the brain did not reveal any underlying vascular lesions.

**Figure 2:**
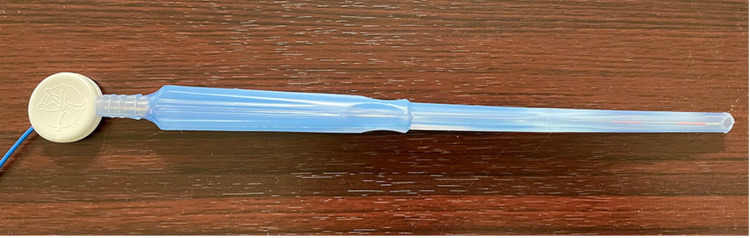
Yankeur straightened and shortened to the length of the Medtronic stylet with the Medtronic stylet inserted into the modified Yankeur

First, the Yankauer was modified by straightening and shortening the length to match that of the Medtronic stylet (Dublin, Ireland), and the Medtronic stylet was then inserted into the modified Yankeur (Figure 2). The patient underwent an approximately 3 cm left craniectomy to allow for the passage of a modified Yankauer into the hematoma cavity, guided by the Stealth navigation system (Medtronic). Once the Yankauer was placed in the hematoma cavity, a vacuum was applied at 50 mmHg and gradually increased in no particular increment until the hematoma was visualized passing through the Yankauer. The pressure did not exceed 520 mmHg. The Yankauer was removed once the hematoma could no longer be aspirated. A drain was then placed in the hematoma cavity. The target was > 90% reduction in the residual hematoma as assessed by the postoperative CT of the brain.

On postoperative Day 1, CT of the brain without contrast showed almost complete evacuation of the hematoma, leaving a 2.2 cm cavity filled with air (Figures 1D-1F). On postoperative Day 3, the patient was extubated. He had dysarthria but was able to say his name and follow simple commands on the left side. He continued to have 0/5 right upper extremity (RUE)/right lower extremity (RLE) motor strength and 5/5 left upper extremity (LUE)/left lower extremity (LLE) motor strength. The patient was discharged on postoperative Day 16 with persistent dysarthria and 0/5 RUE/RLE motor strength.

**Figure 3:**
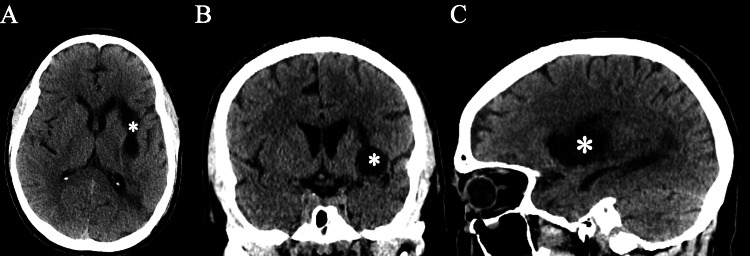
CT of the brain - axial (A), coronal (B), and sagittal (C) cuts approximately two months post-operation demonstrating encephalomalacia (*) of the basal ganglia, involving the globus pallidus and the internal capsule complex

At the one-month follow-up appointment, the patient showed significant improvement in his dysarthria with 1/5 RUE/RLE and 5/5 LUE/LLE in motor strength. The follow-up CT of the brain demonstrated encephalomalacia of the basal ganglia, involving the globus pallidus and the internal capsule complex (Figure 3). At the three-month follow-up appointment, the patient showed drastic improvement. RUE/RLE motor strength was 4/5 and LUE/LLE motor strength was 5/5. The patient was able to walk with a walker, and his speech was fluent with minimal dysarthria.

## Discussion

The poor prognosis of ICH can be attributed to its pathophysiological responses consisting of primary and secondary brain injury. The primary brain injury is mediated by the mechanical compression caused by the hematoma, which may increase intracranial pressure, compress the brain, and consequently lead to brain edema and herniation as shown in our patient. The processes involved in secondary brain injury are multifactorial and yet to be fully understood. Inflammatory mechanisms secondary to the accumulation of neurotoxins and the consequential activation of microglia leading to the release of interleukins (IL-1β, IL-6, IL-12, IL-23) and tumor necrosis factor-alpha (TNF- α) are thought to be the main pathologic process [6-8]. In addition, the divalent iron released from the lysed erythrocytes reacts with lipid to produce lipid reactive oxygen species (ROS), whose accumulation can lead to a type of programmed cell death called ferroptosis [9]. Lastly, the thrombin and fibrin derived from hemorrhagic blood can disrupt the blood brain barrier (BBB) through phosphorylation of Src kinases and microglial activation, leading to disruption of homeostasis of water, electrolytes, and neurotoxic factors, further contributing to neuronal damage [10].

To prevent or minimize primary and secondary brain injury, early hematoma evacuation is an essential component of ICH management. A meta-analysis of many prospective randomized controlled trials studying the timing of ICH surgical intervention suggested that early operation (within 8 hours of ictus) leads to improved outcomes with an odds ratio of 0.59 (95% CI 0.42, 1.84; p=0.003) [11]. However, a standardized ICH evacuation technique is yet to be determined, as studies lack conclusive results. For example, the STICH (surgical treatment for intracerebral hemorrhage) I and II trials proved craniotomy to be an ineffective alternative to the conservative medical treatment of ICH [12-13]. Most other evacuation techniques, including craniectomy, simple aspiration, and endoscopic evacuation, also showed mixed results and lacked sufficient research [14].

In recent decades, minimally invasive ICH evacuation methods have developed, and initial trials showed promising results; however, the MISTIE III (Minimally Invasive Surgery Plus Alteplase for ICH Evacuation) trial failed to show any advantage over standard medical care and elucidated the need for procedure amelioration [15]. In our case, we have developed and used a novel technique called stereotactic vacuum-assisted MIS aspiration of hematoma to evacuate the patient’s ICH, and it showed to be very effective with excellent patient outcomes.

The patient shown here suffered severe ICH, and his/her predicted outcome, as measured by multiple valid assessment tools, was unfavorable. First, the ICH was 45 cm^3^ in volume, completely obscuring the basal ganglia structure on the MRI image, and such hematoma volume combined with his low level of consciousness was found to be associated with a predicted 30-day mortality of 74% [16]. Second, the ICH score, which considers the GCS, ICH volume, origin and location, and age, was 3/6, which is associated with a 30-day mortality rate of 72% [17]. Lastly, the functional outcome in patients with a primary intracerebral hemorrhage (FUNC) score, which helps predict the likelihood of functional independence at 90 days, was 6/9, which is associated with a 1% to 20% chance of recovery to functional independence [18]. Despite these poor predicted outcomes, the patient demonstrated excellent recovery, being functionally independent on his 90-day follow-up. The novel MIS technique of hematoma evacuation described here can be performed with ease, using standard equipment widely available at most hospitals. Future research should aim to compare the effectiveness of this technique to other evacuation techniques and to help establish clear guidelines for ICH evacuation and management.

## Conclusions

Despite major advancements in clinical neurology and neurosurgery, ICH continues to carry a significant mortality rate. This is partially due to the lack of a standardized treatment protocol, notably in the techniques used for ICH evacuation. In our case, the novel MIS stereotactic vacuum-assisted aspiration used for hematoma evacuation demonstrated promising results. This technique can be performed with ease, using standard equipment widely available at most hospitals. This allows for easy application and facilitates further assessment of its effectiveness in other hospital settings. Future endeavors should focus on assessing and comparing its effectiveness to preceding techniques and sharing efforts to establish a standardized protocol for ICH treatment and management.
